# Validation of the Children’s Eating Behavior Questionnaire in 5 and 6 Year-Old Children: The GUSTO Cohort Study

**DOI:** 10.3389/fpsyg.2019.00824

**Published:** 2019-04-11

**Authors:** Phaik Ling Quah, Lisa R. Fries, Mei Jun Chan, Anna Fogel, Keri McCrickerd, Ai Ting Goh, Izzuddin M. Aris, Yung Seng Lee, Wei Wei Pang, Iccha Basnyat, Hwee Lin Wee, Fabian Yap, Keith M. Godfrey, Yap-Seng Chong, Lynette P. C. Shek, Kok Hian Tan, Ciaran G. Forde, Mary F. F. Chong

**Affiliations:** ^1^Singapore Institute for Clinical Sciences, Agency for Science, Technology and Research, Singapore, Singapore; ^2^Nestlé Research, Lausanne, Switzerland; ^3^Clinical Nutrition Research Center, Singapore Institute for Clinical Sciences, Agency for Science, Technology and Research, Singapore, Singapore; ^4^Department of Pediatrics, Yong Loo Lin School of Medicine, National University Health System, National University of Singapore, Singapore, Singapore; ^5^Divisions of Pediatric Endocrinology and Diabetes, Khoo Teck Puat-National University Children’s Medical Institute, National University Hospital, National University Health System, Singapore, Singapore; ^6^Department of Obstetrics & Gynaecology, Yong Loo Lin School of Medicine, National University of Singapore – National University Health System, Singapore, Singapore; ^7^Saw Swee Hock School of Public Health, National University of Singapore, Singapore, Singapore; ^8^Department of Pharmacy, Faculty of Science, National University of Singapore, Singapore, Singapore; ^9^Department of Pediatrics, KK Women’s and Children’s Hospital, Singapore, Singapore; ^10^Medical Research Council Lifecourse Epidemiology Unit, National Institute for Health Research Southampton Biomedical Research Centre, The University of Southampton, University Hospital Southampton NHS Foundation Trust, Southampton, United Kingdom; ^11^Department of Obstetrics and Gynaecology, KK Women’s and Children’s Hospital, Singapore, Singapore; ^12^Department of Physiology, Yong Loo Lin School of Medicine, National University of Singapore, Singapore, Singapore

**Keywords:** children’s eating behaviors, BMI z-score, confirmatory factor analysis, exploratory factor analysis, growing up in Singapore toward healthy outcomes

## Abstract

Revised subscales of the Children’s Eating Behavior Questionnaire (CEBQ) have been proposed to be more appropriate for assessing appetitive traits in Singaporean 3 year-olds, but the CEBQ has not yet been validated in older children in this population. The current study aimed to validate the CEBQ at ages 5 (*n* = 653) and 6 (*n* = 449) in the ethnically diverse GUSTO cohort. Confirmatory factor analysis (CFA) examined whether the established eight-factor model of the CEBQ was supported in this sample. Overall, the CFA showed a poor model fit at both ages 5 and 6. At both ages 5 and 6, an exploratory factor analysis revealed a six-factor structure: food fussiness, enjoyment of food, slowness in eating, emotional undereating, emotional overeating and desire to drink. Cronbach’s alpha estimates ranged from 0.70 to 0.85 for all subscales. Criterion validity was tested by correlating subscales with the weight status of 6 years of age. At age 5 and 6, lower scores of slowness of eating while higher scores of enjoyment of food was associated with child overweight. At age 6, higher scores of desire to drink was also associated child overweight. In conclusion, a revised six factor-structure of the CEBQ at ages 5 and 6 were more appropriate for examining appetitive traits in this sample.

## Introduction

Individual differences in appetitive traits may determine how children learn to regulate their food intake, which eventually determines their weight gain later on in life (Fisher and Birch, 2002; Jansen et al., 2003; Wardle, 2007). The preschool years are a critical period when distinct eating behavior traits are formed, and when obesity interventions are most effective (Reinehr et al., 2010; Danielsson et al., 2012), making this a sensitive window for shaping healthy eating behaviors (Ashcroft et al., 2008; Svensson et al., 2011).

The Children’s Eating Behavior Questionnaire (CEBQ) (Wardle et al., 2001) is a parental-report instrument, developed to measure four food-approach subscales (food responsiveness, enjoyment of food, emotional overeating, and desire to drink), and four food-avoidant subscales (slowness in eating, satiety responsiveness, food fussiness, and emotional undereating) using 35 items. It was validated in the United Kingdom, in a sample of children with a mean age of 5 years with the use of directly observed measures of eating behavior (Carnell and Wardle, 2007), and has since been widely used in other countries (Croker et al., 2011; van Jaarsveld et al., 2011; Fuemmeler et al., 2013).

However, subsequent validation studies in other Western countries and in Asian countries have shown mixed results, with some replicating the original subscales (Mallan et al., 2013; Domoff et al., 2015), while others could not (Sleddens et al., 2008; Santos et al., 2011; Svensson et al., 2011; Cao et al., 2012; Quah et al., 2017). For studies that have failed to replicate the original subscales, exploratory factor analysis (EFA) was used to generate revised subscales that were more culturally relevant to Western countries like Sweden (Svensson et al., 2011), the Netherlands (Sleddens et al., 2008), Chile (Santos et al., 2011), and to Asian countries such as China (Cao et al., 2012), and Singapore (Quah et al., 2017).

Other than cultural factors affecting the interpretation of the items of this questionnaire, the emergence of different subscales across studies may vary depending on the age at which these eating behaviors were captured (Wardle et al., 2001; Ashcroft et al., 2008; Sleddens et al., 2008; Svensson et al., 2011). There is evidence suggesting that eating behaviors may still be changing and developing in children between ages 1–6 years (Dovey et al., 2008; Svensson et al., 2011).

We have previously demonstrated that among Singaporean preschoolers that CEBQ administered at children aged 3 year old was a poor fit to the original questionnaire by Wardle et al. (2001). The factor structure differed from the original, such that items from the enjoyment of food, food responsiveness and food fussiness subscales, and items from the food responsiveness and emotional eating items were merged as new subscales (Quah et al., 2017). However, as eating behaviors tend to stabilize at later ages (Wardle et al., 2001), we hypothesize that the original 8-factor CEBQ administered to our Singapore population may be a better fit in the older children. In the current study, we aim to examine the fit of the original 8-factor CEBQ 2–3 years later in children aged 5 and 6 years of age.

## Materials and Methods

### Study Design and Participants

We obtained data from the Growing Up in Singapore Toward healthy Outcomes (GUSTO) Study^1^. Detailed information on study design and measurements were previously published (Soh et al., 2014). In this study, the main participants were pregnant Chinese, Malay, and Indian women recruited at 14 weeks of gestation from 2 major public maternity units of Kandang Kerbau Women’s and Children’s Hospital (KKH) and the National University Hospital (NUH) in Singapore from June 2009 to September 2010. Women excluded from the study were those on chemotherapy or psychotropic drugs, or those with type 1 diabetes. Of 3751 women screened, 2034 met eligibility criteria, 1247 were recruited and 1152 women had naturally conceived singleton pregnancies. This study was carried out in accordance with the recommendations from the National Healthcare Group Domain Specific Review Board and the SingHealth Centralized Institutional Review Board with written informed consent from all the mothers. All the mothers gave written informed consent in accordance with the Declaration of Helsinki. The protocol was approved by the National Healthcare Group Domain Specific Review Board and the SingHealth Centralized Institutional Review Board.

### Measures

#### Maternal and Child Characteristics

We collected data on maternal ethnicity, educational level, household income, marital status, and maternal age from participants at the recruitment visit. Maternal pre-pregnancy BMI was derived from self-reported pre-pregnancy weight, and standing height measured with the stadiometer (SECA model 213). Information on child sex and birth order was obtained from obstetric records. The weight of the child at 6 years of age was measured using a calibrated digital scale to the nearest 10 g, and standing height was measured with the use of a stadiometer (SECA model 813; SECA Corp.). For reliability, all measurements were taken in duplicates. Child BMI was calculated as weight divided by the square of height. Based on WHO Child Growth Standards 2007, age and sex-adjusted BMI z-scores were derived using the WHO Anthro software (Version 3.2.2) (de Onis et al., 2007). Using the Center for Disease Control (CDC) guidelines, overweight subjects are defined as BMI z-scores at or above the 85th percentile, normal weight as 5th percentile to less than the 85th percentile, and underweight subjects as those below the 5th percentile (Kuczmarski et al., 2000).

#### Children’s Eating Behavior Subscales at 5 and 6 Years

The original CEBQ was intended to capture eight eating behavior subscales (Wardle et al., 2001): Satiety Responsiveness; Slowness in Eating; Food Fussiness; Food Responsiveness; Enjoyment of Food; Desire to Drink; Emotional Undereating and Emotional Overeating. It was completed by the mother in English, which is the administrative language in Singapore, when her child turned 5 and 6 years old.

### Statistical Analysis

As with our previous study on the validation of the CEBQ in children aged 3, the CFA was applied in this study to test the adequacy of fit of the 35-item, 8-factor CEBQ model hypothesized in the original development paper by Wardle et al. (2001). In children aged 3, the statistical methods of the CFA and EFA, revealed a revised seven-factor structure of eating behavior subscales that were more suited for our population (Quah et al., 2017). To enable comparisons with our previous study (Quah et al., 2017) and others (Sleddens et al., 2008; Svensson et al., 2011), we have adopted similar data analytical methods, and have analyzed our data sequentially as follows: (i) adequacy of fit of the CEBQ at ages 5 and 6 years to original model using the CFA. (ii) if required, modification indices will be explored and parameters will be freed to improve the model fit. (iii) if modifications fail to improve the model fit, an EFA approach will be adopted to generate revised subscales for the questionnaire at each time point.

The model fit was evaluated using several fit indices: RMSEA, SRMR, CFI, and TLI. Smaller values for RMSEA (ideally < 0.06) and SRMR (<0.08), while larger values for CFI and TLI (ideally > 0.90) are indicative of acceptable model fit of the data (Hu and Bentler, 1999). A chi-square statistic was used to test the overall model fit, where non-significance (*p* > 0.05) suggests the model fits the data (Hu and Bentler, 1999). The EFA was run using the principal component factor method, and the number of factors to retain was determined using parallel analysis. Factors extracted were retained for further analysis when eigenvalues from actual data are larger than parallel eigenvalues from random data. This method is a more reliable than solely depending on eigenvalue scores generated by factor analytic processes alone, and was chosen to minimize over-extraction of factors (Horn, 1965). The varimax rotation was still applied for easier interpretation, and a factor loading cutoff of >0.35 was applied (Kim and Mueller, 1978; Kline, 1994). New variables (estimated scores) were generated for further analysis by the default regression method (Thompson scoring) (Thomson, 1951).

Of 1152 women with naturally conceived singleton pregnancy, and who met the eligibility criteria, *n* = 668 subjects filled out the questionnaire at year 5 and *n* = 468 at year 6. Subjects who did not participate in the study were the ones who did not answer the questionnaire, twins and drop-outs of the study. Out of all the subjects who attempted the questionnaires, 2.3% (*n* = 15) of the subjects at year 5, and 4.9% (*n* = 23) at year 6, had missing data from not completing the entire questionnaire. We had a subgroup of *n* = 375 subjects who answered the questionnaires at both ages 5 and 6 years that was used to conduct sensitivity analysis. All values were assumed to be missing at random based on the Little Missing Completely at Random (MCAR) test (*p*-value > 0.05). Based on this assumption and the low percentage of missing data, we chose to apply a listwise deletion to the subjects with missing data (Kang, 2013).

The distribution of maternal and infant characteristics between subjects who completed the questionnaires at years 5, 6 and at both time points were compared using the chi-square test for categorical outcome variables, and ANOVA for continuous outcomes to determine if there were significant differences across the groups. Internal consistency of the subscales was determined using Cronbach’s alpha coefficient, where a value greater than 0.7 was considered to be acceptable (Tavakol and Dennick, 2011). Criterion-related validity was assessed by using a one-way ANCOVA (analysis of covariance) to examine the associations between the eating behavior subscales and weight status (overweight, normal weight, and underweight) controlling for maternal ethnicity. A stratified analysis was also conducted to examine these associations by ethnic groups. All analyses were performed using STATA version 14.1 (StataCorp LP, United States). A 2-sided *p* < 0.05 was considered to be statistically significant.

## Results

### Participants of the Study

Characteristics of the participants who completed the questionnaire at age 5 (*n* = 653) and age 6 (*n* = 445) were included in Supplementary Table 1. There were no significant differences in the characteristics between the two groups. On average, mothers were 30 years old, of post-secondary or tertiary educational status, had an average household income of S$2000-5999, a pre-pregnancy BMI of 26.5 and a majority was married. Approximately half the children were boys and were not first born, and the average BMI z-score at age 6 years was within the normal range of 0.06 to 0.08. There were approximately 16% of children who were overweight, 5% who were underweight and 80% with normal weight statuses. The characteristics of the subset with overlapping subjects (*n* = 375) at both time points did not significantly differ from these two groups (Supplementary Table 1).

### Confirmatory Factor Analysis (CFA) and Exploratory Factor Analysis (EFA) of the Children’s Eating Behavior Questionnaire (CEBQ) in Children Aged 5 and 6 years

The model was a poor fit at age 5 years according to the CFA goodness-of-fit indices: χ^2^(540) = 2890.2, *p* < 0.001, *RMSEA* = 0.081 (*PCLOSE* < 0.001), *SRMR* = 0.233, *CFI* = 0.780, and *TLI* = 0.758 (Hu and Bentler, 1999). Modification indices followed by freeing of parameters did not improve the model fit. We then proceeded to use EFA as a data driven approach to generate revised eating behavior subscales. The parallel analysis recommended 6 factors to be retained, and items were retained with factor loading scores of 0.35 or greater. Of the six factors, two subscales, food fussiness and desire to drink, maintained their original items, whereas four had revised items lists (Table 1). In the revised enjoyment of food subscale, four items from the enjoyment of food subscale loaded together with four items from the food responsiveness subscale, and one item from the satiety responsiveness subscale (“My child has a big appetite”). In the revised slowness in eating subscale, four items from the slowness in eating subscale loaded together with two items from the satiety responsiveness subscale. In the revised emotional undereating subscale, four items from the emotional undereating subscale loaded together with two items from the satiety responsiveness subscale. In the revised emotional overeating subscale, one item from the food responsiveness subscale loaded together with all four items from the emotional overeating subscale.

**Table 1:** Factor loadings for all items of the Children’s Eating Behavior Questionnaire (CEBQ) at 5 years and Cronbach’s alpha scores for each factor structure (*n* = 653).

Items^a^	Factors determined through factor analysis^b^						Original subscale^b^	Cronbach’s alpha
			
	FF	EF	SE	EUE	EOE	DD		
**Factor 1; 12.2% variance**								
My child refuses new foods at first	0.774						FF	0.85
My child enjoys tasting new foods (R)	0.831						FF	
My child enjoys a wide variety of foods (R)	0.702						FF	
My child is difficult to please with meals	0.536						FF	
My child is interested in tasting food s/he hasn’t tasted before (R)	0.806						FF	
My child decides that s/he doesn’t like a food, even without tasting it	0.669						FF	
**Factor 2; 11.6% variance**								
My child loves food		0.516					EF	0.82
My child is interested in food		0.547					EF	
My child looks forward to mealtimes		0.593					EF	
My child enjoys eating		0.578					EF	
My child is always asking for food		0.628					FR	
If allowed to, my child would eat too much		0.527					FR	
Given the choice, my child would eat most of the time		0.509					FR	
Even if my child is full up s/he finds room to eat his/her favorite food		0.645					FR	
My child has a big appetite		0.630					SR	
**Factor 3; 10.8% variance**								
My child finishes his/her meal quickly (R)			0.658				SE	0.80
My child eats slowly			0.759				SE	
My child leaves food on his/her plate at the end of a meal			0.409				SR	
My child takes more than 30 min to finish a meal			0.780				SE	
My child gets full before his/her meal is finished			0.571				SR	
My child eats more and more slowly during the course of a meal			0.682				SE	
**Factor 4; 9.0% variance**								
My child eats less when angry				0.785			EUE	0.78
My child eats less when s/he is tired				0.740			EUE	
My child eats more when she is happy				0.481			EUE	
My child eats less when upset				0.805			EUE	
My child gets full up easily				0.493			SR	
My child cannot eat a meal if s/he has had a snack just before				0.567			SR	
**Factor 5; 7.9% variance**								
2. My child eats more when worried					0.706		EOE	0.74
13. My child eats more when annoyed					0.702		EOE	
15. My child eats more when anxious					0.805		EOE	
27. My child eats more s/he has nothing else to do					0.816		EOE	
34. If given the chance, my child would always have food in his/her mouth					0.439		FR	
**Factor 6; 7.4% variance**								
My child is always asking for a drink						0.697	DD	0.70
If given the chance, my child would drink continuously throughout the day						0.873	DD	
If given the chance, my child would always be having a drink						0.881	DD	


*^a^Items marked with (R) have been reversed scored. There are 35 items in the table as the item with a factor loading score of 0.35 or above. ^*b*^Appetite scale the item was originally intended to measure: EF, enjoyment of food; FF, food fussiness; EOE, emotional over eating; DD, desire to drink; EUE, emotional under eating; SE, slowness in eating; and SR, satiety responsiveness.*

The CEBQ at age 6 years was also not a good fit to the original factor structure according to the CFA goodness-of-fit indices; χ^2^(540) = 2441.6, *p* < 0.001, *RMSEA* = 0.086 (*PCLOSE* < 0.001), *SRMR* = 0.270, *CFI* = 0.749, and *TLI* = 0.742. Similar to year 5, modification indices and freeing up parameters did not improve the model fit. The parallel analysis recommended only 6 factors to be retained from the 7 factors extracted (Table 2). At age 6 years, only two subscales acquired new items: enjoyment of food and emotional overeating. The items loaded onto these two revised subscales were similar to the revised subscales generated at year 5.

**Table 2:** Factor loadings for all items of the CEBQ at 6 years and Cronbach’s alpha scores for each factor structure (*n* = 445).

Items^a^	Factors determined through factor analysis^b^							Original subscale^b^	Cronbach’s alpha
			
	EF	FF	EOE	EUE	SE	DD	SR
**Factor 1; 13.2% variance**								
My child loves food	0.739							EF	0.850
My child has a big appetite	0.718							SR	
My child is interested in food	0.724							EF	
My child is always asking for food	0.590							FR	
Given the choice, my child would eat most of the time	0.465							FR	
My child looks forward to mealtimes	0.657							EF	
My child enjoys eating	0.770							EF	
Even if my child is full up s/he finds room to eat his/her favorite food	0.411							FR	
**Factor 2; 10.2% variance**									
My child refuses new foods at first		0.811						FF	0.850
My child enjoys tasting new foods (R)		0.788						FF	
My child enjoys a wide variety of foods (R)		0.689						FF	
My child is difficult to please with meals		0.483						FF	
My child is interested in tasting food s/he hasn’t tasted before (R)		0.784						FF	
My child decides that s/he doesn’t like a food, even without tasting it		0.700						FF	
**Factor 3; 9.34% variance**									
My child eats more when worried			0.732					EOE	0.810
My child eats more when annoyed			0.785					EOE	
If allowed to, my child would eat too much			0.550					FR	
My child eats more when anxious			0.821					EOE	
My child eats more s/he has nothing else to do			0.550					EOE	
If given the chance, my child would always have food in his/her mouth			0.412					FR	
**Factor 4; 8.36% variance**									
9. My child eats less when angry				0.757				EUE	0.772
11. My child eats less when s/he is tired				0.779				EUE	
23. My child eats more when she is happy				0.538				EUE	
25. My child eats less when upset				0.791				EUE	
**Factor 5; 8.34% variance**									
My child finishes his/her meal quickly (R)					0.729			SE	0.813
My child eats slowly					0.790			SE	
My child takes more than 30 min to finish a meal					0.761			SE	
My child eats more and more slowly during the course of a meal					0.677			SE	
**Factor 6; 7.71 % variance**									
My child is always asking for a drink						0.761		DD	0.817
If given the chance, my child would drink continuously throughout the day						0.857		DD	
If given the chance, my child would always be having a drink						0.874		DD	
**^∗^Factor 7; 5.66% variance**									
My child leaves food on his/her plate at the end of a meal							0.681	SR	0.701
My child gets full before his/her meal is finished							0.655	SR	
My child gets full up easily							0.467	SR	
My child cannot eat a meal if s/he has had a snack just before							0.501	SR	


*^a^Items marked with (R) have been reversed scored. There are 35 items in the table as the item with a factor loading score of 0.35 or above. ^*b*^Appetite scale the item was originally intended to measure: EF, enjoyment of food; FF, food fussiness; EOE, emotional over eating; DD, desire to drink; EUE, emotional under eating; SE, slowness in eating; and SR satiety responsiveness. ^∗^The satiety responsiveness subscale is the seventh factor extracted and was not retained for further analysis.*

The revised subscales generated at both time points were mostly similar when the subset of overlapping *n* = 375 subjects were examined (Supplementary Tables 2, 3). The only differences were seen at year 5, where items from the enjoyment of food subscales loaded together with the food fussiness subscales in this subset but not in the sample of *n* = 653 subjects.

### Internal Reliability and Criterion Validity of the Revised Subscales at Year 5 and 6

The extent to which all the items in a subscale measure the same concept or construct was good based on the Cronbach’s α estimates ranging from 0.70 to 0.85. The variances explained by the EFA models at year 5 and 6 were 58.8 and 56.6%, respectively (Tables 1, 2). Table 3 shows the association between the revised subscales at year 5 and 6 with the child weight status (underweight, normal weight and overweight) at 6 years of age, after adjustment for maternal ethnicity. Higher enjoyment of food and lower slowness in eating subscale scores at year 5 and 6 were associated with the child overweight status at year 6. Higher desire to drink subscale scores at year 6 was also associated to child overweight. Sensitivity analysis conducted in the subset of *n* = 375 subjects show similar significant associations at both 5 and 6 years of age (Supplementary Table 4).

**Table 3:** Comparison between CEBQ revised subscales scores at ages 5 (*n* = 653) and 6 (*n* = 445) years with overweight, normal weight, and underweight status of children at 6 years adjusted for maternal ethnicity using ANCOVA (analysis of covariance).

	BMI z-scores			
	Overweight (*n* = 87)	Normal weight (*n* = 459)	Underweight (*n* = 28)	*P*-value
**CEBQ subscales at year 5**				
Enjoyment of food	0.192	–0.013	–0.417	0.003*
Emotional over eating	0.137	–0.032	–0.203	0.217
Desire to drink	0.140	–0.033	0.005	0.456
Food fussiness	–0.047	0.018	–0.021	0.851
Slowness in eating	–0.498	0.045	0.413	0.001*
Emotional Under Eating	–0.216	0.070	–0.105	0.203

	**BMI z-scores**			
	
	**Overweight (*n* = 66)**	**Normal weight (*n* = 306)**	**Underweight (*n* = 19)**	***P*-value**

**CEBQ subscales at year 6**				
Enjoyment of food	0.298	–0.014	–0.23	0.010*
Emotional over eating	0.162	–0.019	–0.369	0.090
Desire to drink	0.340	–0.047	0.221	0.014*
Food fussiness	0.119	–0.045	0.043	0.486
Slowness in eating	–0.351	0.041	0.186	0.020*
Emotional Under Eating	–0.187	0.066	–0.112	0.640


*^∗^*p* < 0.05 is statistically significant; ANCOVA adjusted for ethnicity.*

## Discussion

In this study, we assessed the validity of the original eight-factor structure of the CEBQ (Wardle et al., 2001) in an Asian Singaporean cohort study in mothers of children aged 5 and 6 years old, and found it to be a poor fit. EFA revealed a revised 6-factor structure when the CEBQ was administered to mothers of children aged 5 and 6 years, respectively, which provided a more appropriate solution for our sample. The cumulative variance explained in our cohort was comparable to studies reported previously (Sleddens et al., 2008; Santos et al., 2011; Svensson et al., 2011). At ages 5 and 6 years old, higher scores of enjoyment of food and lower scores of slowness in eating was associated with overweight status in children aged 6, which provide evidence of criterion validity.

In this study, the enjoyment of food subscale captured in children aged 5 and 6 years was positively associated with overweight in children aged 6. At both time points, the enjoyment of food subscales merged with items from the food responsiveness subscale “My child is always asking for food”, “Given the choice, my child would eat most of the time” and “Even if my child is full up she/he finds room to eat his/her favorite food”, and one item from the satiety responsiveness subscales which was “My child has a big appetite” at both ages 5 and 6. These results from the EFA were consistent with our previous findings at age 3 (Quah et al., 2017), suggesting that the parents in our cohort are consistent with their interpretation of these items across the years. Furthermore, enjoyment of food was the only food approach subscale with scores that were consistently associated with higher BMI at all time points studied in children aged 3 (Quah et al., 2017), 5 and 6 years in our cohort. The direction of this association also concurs with previously reported studies (Sleddens et al., 2008; Viana et al., 2008; Webber et al., 2009; Santos et al., 2011). Interestingly, desire to drink which has been associated with consumption of sugar sweetened beverages (Sweetman et al., 2008) was only associated with child overweight at age 6, but not at younger ages (Quah et al., 2017). This observation could reflect the increase in consumption of sugar sweetened beverages in older children (Sweetman et al., 2008) which was subsequently associated with weight gain.

The items from the emotional eating subscale combined with items from the food responsiveness subscale at ages 5 and 6, which is consistent with our observations at age 3 (Quah et al., 2017). The merging of the emotional overeating subscales with items from the food responsiveness subscales has been reported by two other studies (Sleddens et al., 2008; Svensson et al., 2011). Significant positive associations between emotional overeating and BMI were reported in previous studies when the CEBQ was administered in older children with a mean age of 8 (Viana et al., 2008), 9.5 (Webber et al., 2009), and 9–10 years (Santos et al., 2011), but null associations were reported in our study, and previous studies in children close in age to our sample (mean age: 6.5 years) (Sleddens et al., 2008). These findings suggest that eating triggered by emotional stress may not be accurately or consistently perceived by parents in children at younger ages, and associations with weight might only emerge at later time points.

The slowness in eating subscales captured in children aged 5 and 6 were negatively associated with child overweight at ages 6 years. At year 5, items from the satiety responsiveness subscale “My child leaves food on his/her plate at the end of a meal” and “My child gets full before his/her meal is finished” loaded with the slowness in eating subscale at age 5, consistent with the original study by Wardle et al. (2001). Additionally, slowness in eating was the only food avoidance subscale that was significantly associated with lower BMI z-scores at all time points studied in children aged 3 (Quah et al., 2017), 5 and 6 years in our cohort, and the direction of association concurs with previously reported studies (Sleddens et al., 2008; Viana et al., 2008; Webber et al., 2009; Santos et al., 2011). In a separate study within our cohort, we have also shown that the reported slowness in eating scores at ages 5 and 6 captured using the CEBQ had significant negative correlations with measured eating rates (grams/minute) at similar time points (Fogel et al., 2017). This implies that the parental perception of the child’s slowness in eating concurs with actual eating rates measured in the laboratory, at least in terms of identifying slower eaters.

The emotional undereating subscale at age 5 was the other food avoidance subscale combined with items from the satiety responsiveness subscales. We speculate that parents who perceive a child to be eating less when experiencing negative emotions as a child who is just “not hungry” or “full” all the time. Similar to the emotional overeating subscale, emotional undereating was not associated with child weight outcomes, and previous studies have also seen significant associations between this subscale with lower BMI only in older children (Viana et al., 2008; Webber et al., 2009), but not in younger children (Sleddens et al., 2008).

Interestingly, the current study and our previous findings (Quah et al., 2017) have shown that slowness in eating and enjoyment of food were the only two subscales consistently associated with child BMI at ages 3, and overweight status at age 6 years. This highlights that slowness in eating and enjoyment of food subscales could be the most important eating behavior subscales to use for exploring child weight outcomes in our Singaporean cohort.

### Strengths and Limitations

This study shows that the original 8-factor structure of the CEBQ was not replicated in our population. Instead, at ages 5 and 6 years our study revealed a 6-factor structure that is more valid for use in our population. Our study’s strength lies in the longitudinal collection of the CEBQ from a large multi-ethnic sample, which enabled us to validate the revised CEBQ subscales and compare them across the different time points.

The cultural relevance of the CEBQ for non-western populations have been previously assessed in China (Cao et al., 2012), an ethnically diverse (native Australian, immigrant Chinese, and Indians) setting in Australia (Mallan et al., 2013), and in a multi-ethnic setting of Chinese, Malays, and Indians in Malaysia (Loh et al., 2013). Similar to this study, the CEBQ of these previous studies were shown to be only a moderate or poor fit in the population, and revised factors (Cao et al., 2012; Loh et al., 2013) were also found to be more suitable for use in these populations. Overall, this highlights the value and need for validation studies on eating behavior questionnaires (including assessments of eating self-regulation and eating self-control) in different cultures to provide insight into the complexed influence of culture that may affect the perception of eating behaviors related to overweight/obesity (Ng et al., 2005).

Here, we show that in the multicultural Singaporean context, the CEBQ appears to be a reliable tool for us in different ethnic groups of (predominantly) highly educated mothers.

Our study has limitations which need to be addressed. Firstly, the CEBQ only provides a maternal perception of the child’s eating behavior, so other than the slowness in eating subscale, we still lack objective measures of the other eating behavior subscales. Secondly, the association between the CEBQ subscales with weight status of the children could be confounded by reverse causation (e.g., mothers of heavier children will perceive them as being more food responsive).

### Clinical Implications and Future Directions

A valid and reliable eating behavior construct that is culturally appropriate, and reflects the interpretation by the local population is important for examining hypothesized relationships between eating behaviors and clinical outcomes of interest in our cohort (e.g., child BMI). We also plan to examine modifiable environmental factors (e.g., parenting feeding practices) associated with eating behaviors that might be related to child overweight such as the subscales of enjoyment of food and slowness in eating. These subscales will also be essential for examining associations with subsequent outcomes of child dietary intakes and growth. Our future studies shall also aim to establish the usefulness of the CEBQ across samples that differ by ethnicity.

## Conclusion

From our validation study, a revised six factor model may be more relevant in measuring children’s eating behaviors at ages 5 and 6 years in our Singapore population. Furthermore, it supports the use of the revised slowness in eating and the enjoyment of food subscales in this questionnaire for examining relationships with child BMI outcomes. Our future studies shall also aim to establish the usefulness of the CEBQ across samples that differ by ethnicity.

## Ethics Statement

This study was carried out in accordance with the recommendations from the National Healthcare Group Domain Specific Review Board and the SingHealth Centralized Institutional Review Board with written informed consent from all subjects. All subjects gave written informed consent in accordance with the Declaration of Helsinki. The protocol was approved by the National Healthcare Group Domain Specific Review Board and the SingHealth Centralized Institutional Review Board.

## Author Contributions

All authors were involved in all parts of the study and approved the final manuscript. FY, KMG, LPCS, KHT, and Y-SC designed and led the GUSTO cohort study. PLQ, MFFC, LRF, and CGF designed the study. PLQ contributed to the statistical analysis and writing of the manuscript. IB, AF, KM, and HLW provided intellectual contribution to the write-up of the manuscript and advice on the statistical analysis. MJC, ATG, and WWP were involved in the collection and the processing of the Children’s Eating Behavior Questionnaire. IMA and YSL contributed to the anthropometric data collection and the generation of the BMI *z*-score. PLQ, CGF, LRF, and MFFC were responsible for finalizing the manuscript.

## Conflict of Interest Statement

KMG and Y-SC have received reimbursement for speaking at conferences sponsored by companies selling nutritional products. These authors are part of an academic consortium that has received research funding from commercial affiliations such as Abbott Nutrition, Nestec, and Danone. LRF is an employee of Nestec SA, working at the Nestlé Research Center. CGF has received reimbursement for speaking at conferences sponsored by companies selling nutritional products, serves on the scientific advisory council for Kerry Taste and Nutrition, and is part of an academic consortium that has received research funding from Abbott Nutrition, Nestec, and Danone. The remaining authors declare that the research was conducted in the absence of any commercial or financial relationships that could be construed as a potential conflict of interest.

## Footnotes

1 www.clinicaltrials.gov, NCT01174875

## Abbreviations

BMI z-score: body mass index z-score
CEBQ: children eating behavior questionnaire
CFA: confirmatory factor analysis
CFI: comparative fit index
EFA: exploratory analysis
RMSEA: root mean square error of approximation
SRMR: standardized root mean square residual
TLI: tucker lewis index
