# Supplementary material for: Validation of the Children’s Eating Behavior Questionnaire in 5 and 6 Year-Old Children: The GUSTO Cohort Study
Source: Front Psychol. 2019 Apr 11;10:824. doi: 10.3389/fpsyg.2019.00824 (PMC6470280; doi:10.3389/fpsyg.2019.00824)
Supplement: Supplementary file 1 [file Table_1.DOCX]

Supplementary Table 1: Maternal and child characteristics of those who completed the Children’s Eating Behavior Questionnaire (CEBQ) at ages 5(n=653), 6(n=445) years, and subjects who completed the questionnaire at both timepoints(n=375).

|  |  | **Completed questionnaire**  **at year 5**  **(n=653)** | | | **Completed questionnaire**  **at year 6**  **(n=445)*** | | **Completed questionnaire**  **at year 5 and 6**  **(n=375)#** | | | ***P* value** |
| --- | --- | --- | --- | --- | --- | --- | --- | --- | --- | --- |
| **Maternal characteristics** |  | N/Mean | %/SD |  | N/Mean | %/SD | N/Mean | %/SD |  |  |
|  |  |  |  |  |  |  |  |  |  |  |
| **Ethnicity** |  |  |  |  |  |  |  |  |  | 0.698 |
| Chinese |  | 323 | 49.3 |  | 213 | 47.9 | 176 | 46.8 |  |  |
| Malay |  | 193 | 29.7 |  | 148 | 33.3 | 127 | 33.9 |  |  |
| Indian |  | 137 | 21.0 |  | 84 | 18.8 | 72 | 19.3 |  |  |
|  |  |  |  |  |  |  |  |  |  |  |
| **Education** |  |  |  |  |  |  |  |  |  | 0.934 |
| None/primary/secondary |  | 165 | 25.6 |  | 111 | 25.0 | 98 | 26.5 |  |  |
| Post-secondary/tertiary |  | 479 | 74.4 |  | 322 | 71.7 | 272 | 73.5 |  |  |
|  |  |  |  |  |  |  |  |  |  |  |
| **Household income** |  |  |  |  |  |  |  |  |  | 1.000 |
| S$0-1999 (low) |  | 89 | 14.4 |  | 64 | 15.2 | 55 | 15.4 |  |  |
| S$2000-5999 (average) |  | 334 | 54.9 |  | 229 | 54.2 | 195 | 54.4 |  |  |
| >S$6000 (high) |  | 188 | 30.7 |  | 129 | 30.6 | 108 | 30.2 |  |  |
|  |  |  |  |  |  |  |  |  |  |  |
| **Marital/Status** |  |  |  |  |  |  |  |  |  | 0.800 |
| Single/Divorced |  | 24 | 3.7 |  | 13 | 3.0 | 12 | 3.3 |  |  |
| Married |  | 613 | 96.3 |  | 414 | 97.0 | 352 | 96.7 |  |  |
|  |  |  |  |  |  |  |  |  |  |  |
| Age(Years) |  | 30.7 | 5.11 |  | 30.6 | 5.20 | 30.5 | 5.10 |  | 0.617 |
|  |  |  |  |  |  |  |  |  |  |  |
| Pre-Pregnancy BMI |  | 26.5 | 4.6 |  | 26.5 | 4.5 | 26.6 | 4.7 |  | 0.897 |
|  |  |  |  |  |  |  |  |  |  |  |
| **Infant Characteristics** |  |  |  |  |  |  |  |  |  |  |
|  |  |  |  |  |  |  |  |  |  |  |
| **Gender** |  |  |  |  |  |  |  |  |  | 0.735 |
| Male |  | 349 | 53.3 |  | 243 | 54.6 | 204 | 54.5 |  |  |
| Female |  | 304 | 46.7 |  | 202 | 45.4 | 170 | 45.5 |  |  |
|  |  |  |  |  |  |  |  |  |  |  |
| **Birth Order** |  |  |  |  |  |  |  |  |  | 0.976 |
| First Child |  | 302 | 46.1 |  | 204 | 46.5 | 177 | 47.3 |  |  |
| Not First Child |  | 349 | 53.9 |  | 235 | 53.5 | 197 | 52.7 |  |  |
|  |  |  |  |  |  |  |  |  |  |  |
| **BMI z-scores at 6 years** |  | 0.06 | 1.46 |  | 0.08 | 1.46 | 0.14 | 1.48 |  | 0.970 |
|  |  |  |  |  |  |  |  |  |  |  |
| **Weight status** |  |  |  |  |  |  |  |  |  |  |
| Underweight |  | 28 | 4.8 |  | 19 | 4.8 | 17 | 5.0 |  | 0.980 |
| Normal weight |  | 459 | 80.0 |  | 306 | 78.3 | 260 | 77.2 |  |  |
| Overweight |  | 87 | 15.2 |  | 66 | 16.9 | 60 | 17.8 |  |  |

Missing data from n=653 participants at year 5 :maternal education(n=12), household income(n=42), marital status(n=16), mother’s age(n=2), mother’s pre-pregnancy BMI (n=23) , BMI z-score(n=91)

*Missing data from n=445 participants at year 6: maternal education(n=16), household income(n=23), marital status(n=18), mothers age(n=15), maternal pre-pregnancy BMI(n=10),birth order(n=6),BMI z-score(n=54)

# Missing data from n=349 participants at year 5 and 6: maternal education(n=5), household income(n=17), marital status(n=11), maternal pre-pregnancy BMI(n=14), BMI z-score(n=38)

Supplementary Table 2: Factor loadings for all items of the Children’s Eating Behavior Questionnaire (CEBQ) at 5 years and Cronbach’s alpha scores for each factor structure n the subset of n=375 subjects.

| **Items^a^** | **Factors determined through factor analysis^b^** | | | | | | | **Original scale^c^** | **Cronbach’s alpha** | |
| --- | --- | --- | --- | --- | --- | --- | --- | --- | --- | --- |
|  | **FF** | **FR** | **SE** | **EUE** | **EOE** | **DD** |  |  |  | |
|  |  |  |  |  |  |  |  |  |  | |
|  |  |  |  |  |  |  |  |  |  | |
| **Factor 1; 14.5% variance** |  |  |  |  |  |  |  |  |  | |
| My child loves food(R) | 0.599 |  |  |  |  |  |  | EF | 0.90 | |
| My child is interested in food(R) | 0.601 |  |  |  |  |  |  | EF |  | |
| My child enjoys eating(R) | 0.572 |  |  |  |  |  |  | EF |  | |
| My child enjoys tasting new foods(R) | 0.854 |  |  |  |  |  |  | FF |  | |
| My child refuses new foods at first | 0.778 |  |  |  |  |  |  | FF |  | |
| My child enjoys a wide variety of foods(R) | 0.740 |  |  |  |  |  |  | FF |  | |
| My child is difficult to please with meals | 0.588 |  |  |  |  |  |  | FF |  | |
| My child is interested in tasting food s/he hasn't tasted before(R) | 0.830 |  |  |  |  |  |  | FF |  | |
| My child decides that s/he doesn't like a food, even without tasting it | 0.687 |  |  |  |  |  |  | FF |  | |
|  |  |  |  |  |  |  |  |  |  |  |
| **Factor 2; 10.4% variance** |  |  |  |  |  |  |  |  |  |  |
| My child has a big appetite |  | 0.561 |  |  |  |  |  | SR | 0.74 |  |
| My child looks forward to mealtimes |  | 0.526 |  |  |  |  |  | EF |  |  |
| My child is always asking for food |  | 0.508 |  |  |  |  |  | FR |  |  |
| Given the choice, my child would eat most of the time |  | 0.519 |  |  |  |  |  | FR |  |  |
|  |  |  |  |  |  |  |  | FR |  |  |
| Even if my child is full up s/he finds room to eat his/her favorite food |  | 0.697 |  |  |  |  |  |  |  |  |
|  |  |  |  |  |  |  |  |  |  |  |
| **Factor 3; 9.9% variance** |  |  |  |  |  |  |  |  |  |  |
| My child finishes his/her meal quickly(R) |  |  | 0.644 |  |  |  |  | SE | 0.78 |  |
| My child eats slowly |  |  | 0.779 |  |  |  |  | SE |  |  |
| My child takes more than 30 minutes to finish a meal |  |  | 0.808 |  |  |  |  | SE |  |  |
| My child eats more and more slowly during the course of a meal |  |  | 0.640 |  |  |  |  | SE |  |  |
| My child gets full before his/her meal is finished |  |  | 0.445 |  |  |  |  | SR |  |  |
|  |  |  |  |  |  |  |  |  |  |  |
|  |  |  |  |  |  |  |  |  |  |  |
| **Factor 4; 9.5% variance** |  |  |  |  |  |  |  |  |  |  |
| My child eats less when angry |  |  |  | 0.726 |  |  |  | EUE | 0.78 |  |
| My child eats less when s/he is tired |  |  |  | 0.714 |  |  |  | EUE |  |  |
| My child eats more when she is happy(R) |  |  |  | 0.464 |  |  |  | EUE |  |  |
| My child eats less when upset |  |  |  | 0.787 |  |  |  | EUE |  |  |
| My child gets full up easily |  |  |  | 0.592 |  |  |  | SR |  |  |
| My child cannot eat a meal if s/he has had a snack just before |  |  |  | 0.590 |  |  |  | SR |  |  |
|  |  |  |  |  |  |  |  |  |  |  |
| **Factor 5; 8.9% variance** |  |  |  |  |  |  |  |  |  |  |
| My child eats more when worried |  |  |  |  | 0.705 |  |  | EOE | 0.80 |  |
| My child eats more when annoyed |  |  |  |  | 0.794 |  |  | EOE |  |  |
| If allowed to, my child would eat too much |  |  |  |  | 0.526 |  |  | EOE |  |  |
| My child eats more when anxious |  |  |  |  | 0.825 |  |  | EOE |  |  |
| My child eats more s/he has nothing else to do |  |  |  |  | 0.512 |  |  | FR |  |  |
| If given the chance, my child would awalys have food in his/her mouth |  |  |  |  | 0.467 |  |  | FR |  |  |
|  |  |  |  |  |  |  |  |  |  |  |
| **Factor 6; 7.5% variance** |  |  |  |  |  |  |  |  |  |  |
| My child is always asking for a drink |  |  |  |  |  | 0.705 |  | DD | 0.79 |  |
| If given the chance, my child would drink continuously throughout the day |  |  |  |  |  | 0.841 |  | DD |  |  |
| If given the chance, my child would always be having a drink |  |  |  |  |  | 0.863 |  | DD |  |  |
|  |  |  |  |  |  |  |  |  |  |  |
|  |  |  |  |  |  |  |  |  |  |  |

a Items marked with (R) have been reversed scored. There are 35 items in the table as the item with a factor loading score of 0.35 or above.

^c^ Appetite scale the item was originally intended to measure: EF,’ enjoyment of food’; FF,’ food fussiness’; EOE, ‘ Emotional Over Eating’; DD, ‘desire to drink’;

EUE, ‘ Emotional Under Eating’; SE‘slowness in eating’; ’SR ‘ satiety responsiveness’

Supplementary Table 3: Factor loadings for all items of the Children’s Eating Behavior Questionnaire (CEBQ) at 6 years and Cronbach’s alpha scores for each factor structure in the subset of n=375 subjects.

| **Items^a^** |  | **Factors determined through factor analysis^b^** | | | | | | | | | |  | | |  | | |  |  |
| --- | --- | --- | --- | --- | --- | --- | --- | --- | --- | --- | --- | --- | --- | --- | --- | --- | --- | --- | --- |
|  | **FR** | | **FF** | **EOE** | | **SE** | | **EUE** | **DD** | **SR** | | | **Original Scale^c^** | | **Cronbach’s alpha** | | |  |  |
|  |  | |  |  | |  | |  |  |  | | |  | |  | | |  |  |
|  |  | |  |  | |  | |  |  |  | | |  | |  | | |  |  |
| **Factor 1; 13.8% variance** |  | |  |  | |  | |  |  |  | | |  | |  | |  |  |  |
| My child loves food | 0.726 | |  |  | |  | |  |  |  | | | EF | | 0.864 | |  |  |  |
| My child is interested in food | 0.729 | |  |  | |  | |  |  |  | | | EF | |  | |  |  |  |
| My child looks forward to mealtimes | 0.688 | |  |  | |  | |  |  |  | | | EF | |  | | | |  |
| My child enjoys eating | 0.772 | |  |  | |  | |  |  |  | | | EF | |  | | | |  |
| My child is always asking for food | 0.622 | |  |  | |  | |  |  |  | | | FR | |  | | | |  |
| If allowed to, my child would eat too much | 0.513 | |  |  | |  | |  |  |  | | | FR | |  | | | |  |
| Given the choice, my child would eat most of the time | 0.521 | |  |  | |  | |  |  |  | | | FR | |  | | | |  |
| Even if my child is full up s/he finds room to eat his/her favorite food | 0.431 | |  |  | |  | |  |  |  | | | FR | |  | | | |  |
| My child has a big appetite | 0.729 | |  |  | |  | |  |  |  | | | SR | |  | | | |  |
|  |  | |  |  | |  | |  |  |  | | |  | |  | | | |  |
|  |  | |  |  | |  | |  |  |  | | |  | |  | | | |  |
| **Factor 2; 10.7% variance** |  | |  |  | |  | |  |  |  | | |  | |  | | | |  |
| My child refuses new foods at first |  | | 0.800 |  | |  | |  |  |  | | | FF | | 0.849 | | | |  |
| My child enjoys tasting new foods(R) |  | | 0.784 |  | |  | |  |  |  | | | FF | |  | | | |  |
| My child enjoys a wide variety of foods(R) |  | | 0.699 |  | |  | |  |  |  | | | FF | |  | | | |  |
| My child is difficult to please with meals |  | | 0.525 |  | |  | |  |  |  | | | FF | |  | | | |  |
| My child is interested in tasting food s/he hasn't tasted before(R) |  | | 0.784 |  | |  | |  |  |  | | | FF | |  | | | |  |
| My child decides that s/he doesn't like a food, even without tasting it |  | | 0.705 |  | |  | |  |  |  | | | FF | |  | | | |  |
|  |  | |  |  | |  | |  |  |  | | |  | |  | | | |  |
|  |  | |  |  | |  | |  |  |  | | |  | |  | | | |  |
| **Factor 3; 8.63% variance** |  | |  |  | |  | |  |  |  | | |  | |  | | | |  |
| My child eats more when worried |  | |  | 0.746 | |  | |  |  |  | | | EOE | | 0.798 | | | |  |
| My child eats more when annoyed |  | |  | 0.805 | |  | |  |  |  | | | EOE | |  | | | |  |
| My child eats more when anxious |  | |  | 0.550 | |  | |  |  |  | | | EOE | |  | | | |  |
| My child eats more s/he has nothing else to do |  | |  | 0.521 | |  | |  |  |  | | | EOE | |  | | | |  |
|  |  | |  |  | |  | |  |  |  | | |  | |  | | | |  |
|  |  | |  |  | |  | |  |  |  | | |  | |  | | | |  |
| **Factor 4; 8.56% variance** |  | |  |  | |  | |  |  |  | | |  | |  | | | |  |
| My child finishes his/her meal quickly(R) |  | |  |  | | 0.734 | |  |  |  | | | SE | | 0.819 | | | |  |
| My child eats slowly |  | |  |  | | 0.765 | |  |  |  | | | SE | |  | | | |  |
| My child takes more than 30 minutes to finish a meal |  | |  |  | | 0.741 | |  |  |  | | | SE | |  | | | |  |
| My child eats more and more slowly during the course of a meal |  | |  |  | | 0.700 | |  |  |  | | | SE | |  | | | |  |
|  |  | |  |  | |  | |  |  |  | | |  | |  | | | |  |
| **Factor 5; 8.26% variance** |  | |  |  | |  | |  |  |  | | |  | |  | | | |  |
| My child eats less when angry |  | |  |  | |  | | 0.752 |  |  | | | EUE | | 0.772 | | | |  |
| My child eats less when s/he is tired |  | |  |  | |  | | 0.775 |  |  | | | EUE | |  | | | |  |
| My child eats more when she is happy(R) |  | |  |  | |  | | 0.539 |  |  | | | EUE | |  | | | |  |
| My child eats less when upset |  | |  |  | |  | | 0.823 |  |  | | | EUE | |  | | | |  |
|  |  | |  |  | |  | |  |  |  | | |  | |  | | | |  |
| **Factor 6; 7.71 % variance** |  | |  |  | |  | |  |  |  | | |  | |  | | | |  |
| My child is always asking for a drink |  | |  |  | |  | |  | 0.743 |  | | | DD | | 0.818 | | | |  |
| If given the chance, my child would drink continuously throughout the day |  | |  |  | |  | |  | 0.869 |  | | | DD | |  | | | |  |
| If given the chance, my child would always be having a drink |  | |  |  | |  | |  | 0.875 |  | | | DD | |  | | | |  |
|  |  | |  |  | |  | |  |  |  | | |  | |  | | | |  |
|  |  | |  |  | |  | |  |  |  | | |  | |  | | | |  |
| **Factor 7 ; 5.66% variance** |  | |  |  | |  | |  |  |  | | |  | |  | | | |  |
| My child leaves food on his/her plate at the end of a meal |  | |  |  | |  | |  |  | 0.672 | | | SR | | 0.706 | | | |  |
| My child gets full before his/her meal is finished |  | |  |  | |  | |  |  | 0.674 | | | SR | |  | | | |  |
| My child gets full up easily |  | |  |  | |  | |  |  | 0.503 | | | SR | |  | | | |  |
| My child cannot eat a meal if s/he has had a snack just before |  | |  |  | |  | |  |  | 0.458 | | | SR | |  | | | |  |
|  |  | |  |  | |  | |  |  |  | | |  | |  | | | |  |
|  |  | |  |  | |  | |  |  |  | | |  | |  | | | |  |
|  |  | |  |  | |  | |  |  |  | | |  | |  | | | |  |
|  |  | |  |  | |  | |  |  |  | | |  | |  | | | |  |
|  |  | |  |  |  | |  | | |  |  | | |  | |  | | | |

a Items marked with (R) have been reversed scored. There are 35 items in the table as the item with a factor loading score of 0.35 or above.

^c^ Appetite scale the item was originally intended to measure: EF,’ enjoyment of food’; FF,’ food fussiness’; EOE, ‘ Emotional Over Eating’; DD, ‘desire to drink’;

EUE, ‘ Emotional Under Eating’; SE‘slowness in eating’; ’SR ‘ satiety responsiveness’

Supplementary Table 4: Associations between CEBQ revised subscales scores at ages 5 and 6 years with overweight, normal weight and underweight status of children at 6 years adjusted for maternal ethnicity using ANCOVA(analysis of covariance) in the subset of n=375 subjects.

|  |  | **BMI z-score** |  |  |
| --- | --- | --- | --- | --- |
|  | **Overweight**  **(n=60)** | **Normal weight**  **(n=260)** | **Underweight**  **(n=17)** | ***P* value** |
| **CEBQ subscales at year 5** |  |  |  |  |
|  |  |  |  |  |
| Enjoyment of food | 0.022 | 0.047 | -0.638 | 0.030 |
| Emotional over eating | 0.159 | -0.040 | -0.099 | 0.333 |
| Desire to drink | 0.140 | -0.033 | 0.005 | 0.456 |
|  |  |  |  |  |
| Food fussiness | -0.048 | 0.018 | -0.021 | 0.851 |
| Slowness in eating | -0.375 | 0.027 | 0.329 | 0.010 |
| Emotional Under Eating | -0.209 | 0.078 | -0.271 | 0.095 |
|  |  | **BMI z-scores** |  |  |
|  | **Overweight**  **(n=60)** | **Normal weight**  **(n=260)** | **Underweight**  **(n=17)** | ***P* value** |
| **CEBQ subscales at year 6** |  |  |  |  |
|  |  |  |  |  |
| Enjoyment of food | 0.334 | -0.027 | -0.258 | 0.006 |
| Emotional over eating | -0.374 | 0.060 | 0.184 | 0.108 |
| Desire to drink | 0.319 | -0.059 | 0.202 | 0.020 |
|  |  |  |  |  |
| Food fussiness | 0.0277 | -0.016 | 0.036 | 0.938 |
| Slowness in eating | -0.276 | 0.084 | -0.121 | 0.030 |
| Emotional Under Eating | 0.162 | -0.035 | -0.356 | 0.124 |
|  |  |  |  |  |

*p<0.05 is statistically significant; ANCOVA adjusted for ethnicity
